# High-dimensional immunophenotyping reveals immune cell aberrations in patients with undiagnosed inflammatory and autoimmune diseases

**DOI:** 10.1172/JCI169619

**Published:** 2023-12-15

**Authors:** Alisa A. Mueller, Takanori Sasaki, Joshua W. Keegan, Jennifer P. Nguyen, Alec Griffith, Alice M. Horisberger, Thomas Licata, Elizabeth Fieg, Ye Cao, Mehreen Elahee, Kathryne E. Marks, Daimon P. Simmons, Lauren C. Briere, Laurel A. Cobban, J. Carl Pallais, Frances A. High, Melissa A. Walker, Jenny J. Linnoila, Jeffrey A. Sparks, V. Michael Holers, Karen H. Costenbader, Undiagnosed Diseases Network, David A. Sweetser, Joel B. Krier, Joseph Loscalzo, James A. Lederer, Deepak A. Rao

**Affiliations:** 1 Department of Medicine,; 2 Department of Surgery, and; 3 Department of Pathology, Brigham and Women’s Hospital, Boston, Massachusetts, USA.; 4 Center for Genomic Medicine,; 5 Department of Pediatrics, and; 6 Department of Neurology, Massachusetts General Hospital, Boston, Massachusetts, USA.; 7 Department of Medicine, University of Colorado Anschutz Medical Campus, Aurora, Colorado, USA.; 8 The Undiagnosed Diseases Network is detailed in the Supplemental Materials.

**Keywords:** Autoimmunity, Inflammation, Autoimmune diseases, Rheumatology

**To the Editor:** Tools to identify immune dysregulation in patients with unusual autoimmune or inflammatory conditions remain limited. In this study, we employed mass cytometry immunophenotyping to evaluate PBMCs from 16 patients in the Undiagnosed Diseases Network (UDN) for whom the clinical evaluation team suspected an immune-mediated etiology and genetic sequencing did not yield a diagnostic variant (Supplemental Figure 1A and Supplemental Tables 1 and 2; supplemental material available online with this article; https://doi.org/10.1172/JCI169619DS1). We compared their immunoprofiles to those of comparator patients consisting of 98 patients with no inflammatory condition, 24 patients with systemic lupus erythematous (SLE), and 20 patients with rheumatoid arthritis (RA). We aimed to identify individuals with unique immunologic aberrations that might reveal active pathologic pathways and guide treatment decisions.

Clustering analyses of T and B cell–focused panels demonstrated cell subsets within each major cell lineage (Figure 1A and Supplemental Figures 1, B and C). Patients were identified as outliers if the abundance of a cell cluster — as a proportion of PBMCs or relevant lineage — was the highest value detected and 2-fold greater than the next highest patient, a stringent metric (Figures 1, B and C, and Supplemental Figure 1, D–H). This approach identified outlier features in 5 of 16 UDN patients and 0 of 142 comparators (Figure 1, D–F, and Supplemental Figure 1, I–M).

A striking phenotype was identified in UDN no. 1, a 58-year-old woman with an 8-year history of debilitating global erythroderma, alopecia, and palmar plantar keratoderma who exhibited inadequate response to multiple immunosuppressive agents including methotrexate, tumor necrosis factor inhibition, IL-17A inhibition, IL-4 receptor inhibition, i.v. immunoglobulin, and steroids (Figure 1G, Supplemental Figure 2, A–C, and Supplemental Table 1). She had no family history of immune disease (Supplemental Figure 2A). Serum cytokines including IL-1β, IL-2, IL-6, IL17, and IFN-γ were normal. IL-18 levels were similar to those in RA and SLE patients (Supplemental Figure 2C). Mass cytometry revealed marked expansion of a CD25^hi^CD127^–^ CD4 T cell cluster, a phenotype typical of regulatory T cells (Tregs). This cluster comprised 54% of circulating T cells, 10-fold higher than the mean of other patients (Figure 1, C–F). Outlier analyses also highlighted a unique cluster of CD5^+^Bcl-2^+^ B cells in UDN no. 4 (Supplemental Figure 1, J–M) that further evaluation revealed to be chronic lymphocytic leukemia, and ibrutinib therapy was initiated. Three additional outlier patients were identified: UDN no. 2, UDN no. 3, and UDN no. 5 (Supplemental Figure 1, J–M, and Supplemental Table 1).

The conspicuous increase of CD25^hi^CD127^–^ CD4 T cells in UDN no. 1 prompted us to investigate their potential role in the disease. Flow cytometry confirmed expansion of CD25^hi^CD127^–^ CD4 T cells (Supplemental Figure 2D), and mass cytometry phenotyping showed enrichment of FoxP3, CTLA-4, and Helios, consistent with a Treg phenotype (Supplemental Figure 2E). Immunofluorescence microscopy of skin biopsies confirmed dermal infiltration of FoxP3^+^CD25^+^ cells (Figure 1H and Supplemental Figure 2F). We performed single cell RNA-Seq (scRNA-Seq) of blood CD4 T cells from UDN no. 1 and compared them to CD4 T cells from 28 patients with RA. scRNA-Seq reproduced enrichment of *FOXP3*^+^*IKZF2*^+^ Tregs (Figure 1I and Supplemental Figure 2G) that exhibited an HLA-DR^+^CCR7^–^ active Treg phenotype (Supplemental Figure 2H) (1). Single-cell TCR repertoire analysis of blood T cells and PCR-based clonality studies of blood and skin T cells indicated polyclonality (Supplemental Figure 2I).

We next evaluated whether there were transcriptomic differences between these apparent Tregs in UDN no. 1 and RA Tregs. These aberrant Tregs from UDN no. 1 exhibited increased expression of *TGFB1*, a Treg cytokine, and little expression of inflammatory cytokines including IFN-γ, TNF, or IL-17A (Supplemental Figure 2J). Gene set enrichment analysis showed upregulation of IFN response signaling– and MHC class II–associated genes in Tregs and memory CD4 T cells from UDN no. 1 (Figure 1J and Supplemental Figure 2K) and upregulation of an activation signature (Supplemental Figure 2L). Additionally, the UDN no. 1 Treg-like cells demonstrated impaired suppression of CD4 T cell proliferation (Figure 1K).

Given these findings of T cell dysregulation, the patient was treated with abatacept (CTLA4-Ig), a T cell–directed therapy that can inhibit T cell activation and decrease Treg numbers (2), which reduced the frequency of circulating CD25^hi^CD127^–^ T cells (Figure 1I and Supplemental Figure 2, D and G) and yielded partial clinical response. She was subsequently treated with the JAK inhibitor tofacitinib, which downregulates T cell activation and IFN responses (3). Tregs and memory CD4 T cells from UDN no. 1 showed decreases in IFN and activation signatures (Figure 1L and Supplemental Figure 2M), concordant with substantial clinical improvement.

Cellular and transcriptomic immunophenotyping provide valuable complementary tools to define aberrant immune activation in patients with diseases whose pathogenesis may not be entirely due to a genetic mutation. These analyses demonstrate the potential for immunoprofiling to identify specific immune cell abnormalities and guide clinical care for patients whose disease does not fit into traditional diagnostic categories.

## Supplementary Material

Supplemental data

Unedited blot and gel images

Supporting data values

## Figures and Tables

**Figure 1:**
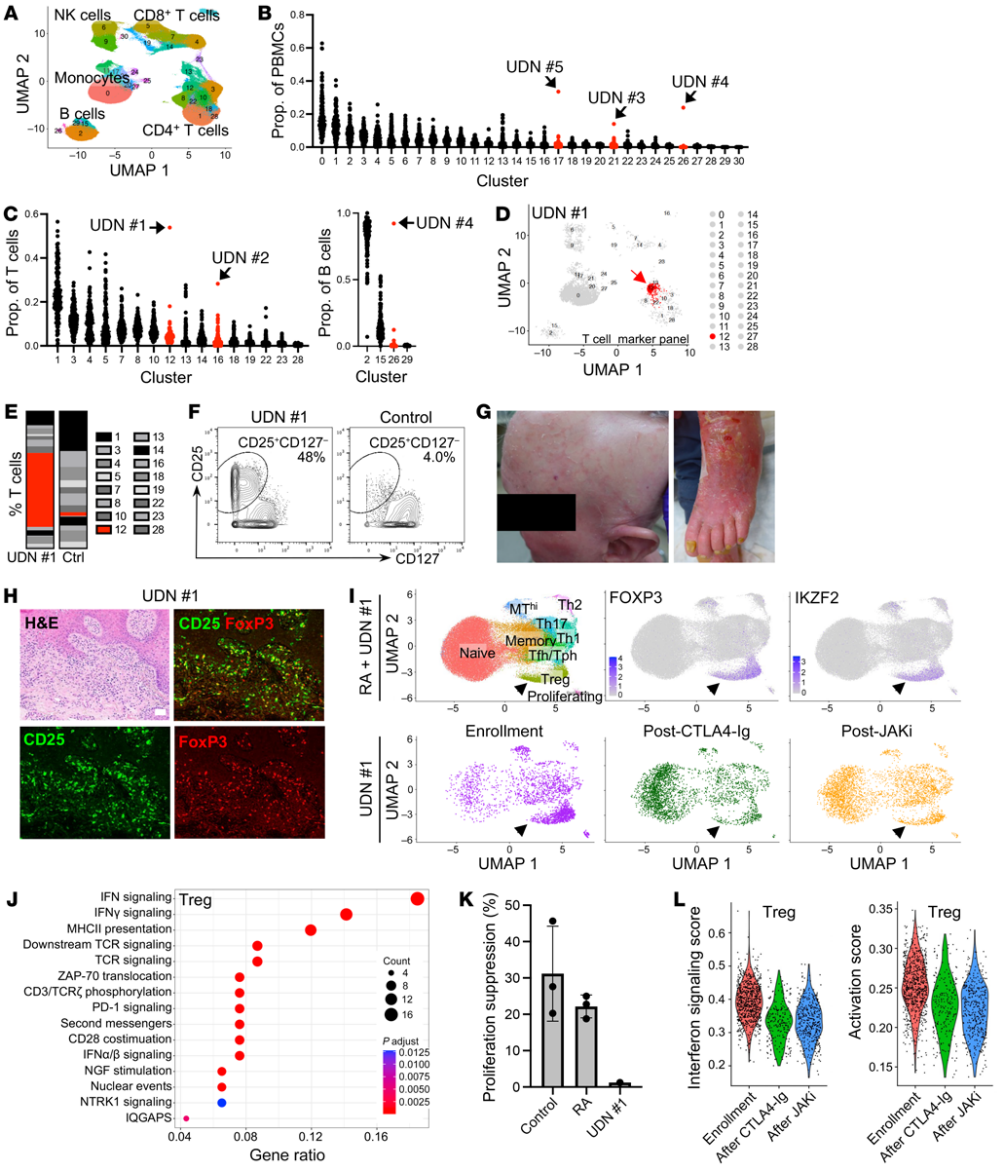
Mass cytometric profiling reveals outlier immune aberrations in undiagnosed patients and dysfunctional Treg expansion in a patient with inflammatory skin disease. (**A**) UMAP of major immune cell populations identified in clustering analyses of mass cytometry T cell marker panel data. (**B** and **C**) For 16 UDN and 142 comparator patients, the cluster frequency as a proportion of total PBMCs (**B**) or the indicated cell populations (**C**). Red, clusters with an outlier. (**D**) UMAP of outlier cluster (arrow) from UDN no. 1. (**E** and **F**) Bar chart (**E**) and biaxial gating plot (**F**) showing frequency of the outlier cluster as a proportion of total T cells for UDN no. 1. (**G**) Photographs depicting alopecia and erythroderma in UDN no. 1. (**H**) H&E and immunofluorescence microscopy of skin biopsies from UDN no. 1; green, CD25; red, FoxP3. Scale bar, 50 µm. (**I**) UMAP of scRNA-Seq data of CD4^+^ T cell populations from patients with RA (n=28) and UDN no. 1 (top). UMAP of CD4^+^ T cell population for UDN no. 1 alone at 3 time points (bottom). Arrows indicate Tregs. (**J**) Top pathways enriched in Tregs from UDN no. 1 compared to patients with RA (n=28). (**K**) Quantification of suppression of CD4^+^ T cell proliferation by Tregs from controls (n=3), patients with RA (n=3), and UDN no. 1. Mean and SD are shown. (**L**) Violin plots illustrating IFN signaling and activation scores in Tregs from UDN no. 1 at enrollment, after CTLA4-Ig therapy, and after JAK inhibitor therapy.

## Version 1 10/24/2023

In-Press Preview

## Version 2 12/15/2023

Electronic publication
