# Supplementary material for: High-dimensional immunophenotyping reveals immune cell aberrations in patients with undiagnosed inflammatory and autoimmune diseases
Source: J Clin Invest. 2023 Dec 15;133(24):e169619. doi: 10.1172/JCI169619 (PMC10721141; doi:10.1172/JCI169619)
Supplement: Supplemental data [file jci-133-169619-s200.pdf]

Supplemental Material

Table of Contents

1. Author Contributions (Page 2)
2. Conflict of Interest (Page 2)
3. Data Availability (Page 2)
4. Acknowledgements (Page 3)
5. Undiagnosed Diseases Network Investigators (Pages 4-5)
6. Materials and Methods (Pages 6-14)
7. Supplemental Figures (Pages 15-18)
8. Supplemental Tables (Pages 19-22)
9. Supplemental References (Pages 23-24)

Author Contributions

D.A.R., J.A.L., A.A.M., T.S., J.B.K., and J.L. conceived of the study. E.F., T.L., A.A.M., L.C.B., L.A.C., D.P.S., J.C.P., F.A.H., M.A.W., J.J.L., J.A.S., V.M.H., K.H.C., D.A.S., J.B.K., J.L., and D.A.R. participated in clinical evaluation and/or patient recruitment and coordination for the study. A.A.M., T.S., J.W.K., J.P.N., A.G., M.E., and K.E.M. designed and/or conducted the experiments. A.A.M. and T.S. performed data analysis. A.M.H. and Y.C. advised on aspects of the data analysis. A.A.M., T.S., and D.A.R. interpreted the data and wrote the manuscript. All authors edited and/or approved the final version of the manuscript.

Conflict of Interest

D.A.R. is co-inventor on a patent using T peripheral helper cells as a biomarker of autoimmune diseases. M.A.W. is author on a patent application (International Patent Application No. PCT/US2021/035951) for a technique to diagnose mitochondrial disorders. J.L. is a scientific cofounder of Scipher Medicine, which develops diagnostic and therapeutic strategies for complex diseases through detailed analysis of individual patient molecular and genomic data sets. The remaining authors declare no competing interests.

Data Availability

Single cell RNA sequencing data and TCR repertoire data will be shared on Synapse upon publication (Project SynID: syn52254335, DOI: https://doi.org/10.7303/syn52254335). Additional data that support the findings of the study are available in the Supporting Data Values files included as part of this manuscript. Data was processed using standard R packages and tools as detailed in the Materials and Methods. Any further data will be made available upon reasonable request to the corresponding author.

Acknowledgements

Research reported in this manuscript was supported by the NIH Common Fund, through the Office of Strategic Coordination/Office of the NIH Director under Award Numbers: U01HG007530 (Harvard Medical School), U01HG007690 (Brigham and Women's Hospital and Massachusetts General Hospital), U01HG007942 (Baylor College of Medicine), and U01HG007943 (HudsonAlpha Institute for Biotechnology). The content is solely the responsibility of the authors and does not necessarily represent the official views of the National Institutes of Health. A.A.M. received support from the Rheumatology Research Foundation Tobé and Stephen E. Malawista, MD, Endowment in Academic Rheumatology and the NIH NIAMS T32 Training Grant (AR007530). D.A.R was supported by a Career Award in Medical Sciences from the Burroughs Wellcome Fund, Clinical Scientist Development Award from Doris Duke Charitable Foundation, and grant support from Lupus Research Alliance, NIH NIAMS K08 (AR072791), and P30 (AR070253). Mass cytometry data on SLE patients was generated with support from Merck Sharp & Dohme. J.J.L. was supported by a NIH NINDS K08 (NS101084) and the McCance Transformative Scholar Award from the Massachusetts General Hospital Department of Neurology. This work was also supported by the Caitlin and Rich Hill Family Fund for Undiagnosed Diseases and the American Institute for Neuro-Integrative Development (AIND) at Mass General Hospital (L.C.B and D.A.S). J.A.S. is supported by the National Institute of Arthritis and Musculoskeletal and Skin Diseases (grant numbers R01 AR080659, R01 AR077607, P30 AR070253, and P30 AR072577), the R. Bruce and Joan M. Mickey Research Scholar Fund, and the Llura Gund Award for Rheumatoid Arthritis Research and Care. The Translational Clinical Research Center at Massachusetts General Hospital was supported by NIH grant number 1UL1TR001102. We thank Dana-Farber/Harvard Cancer Center in Boston, MA, for the use of the Specialized Histopathology Core, which provided histology and immunohistochemistry service. Dana-Farber/Harvard Cancer Center is supported in part by an NCI Cancer Center Support Grant # NIH 5 P30 CA06516.

Undiagnosed Diseases Network Investigators

Maria T. Acosta, Margaret Adam, David R. Adams, Raquel L. Alvarez, Justin Alvey, Laura Amendola, Ashley Andrews, Euan A. Ashley, Carlos A. Bacino, Guney Bademci, Ashok Balasubramanyam, Dustin Baldridge, Jim Bale, Michael Bamshad, Deborah Barbouth, Pinar Bayrak-Toydemir, Anita Beck, Alan H. Beggs, Edward Behrens, Gill Bejerano, Hugo J. Bellen, Jimmy Bennett, Beverly Berg-Rood, Jonathan A. Bernstein, Gerard T. Berry, Anna Bican, Stephanie Bivona, Elizabeth Blue, John Bohnsack, Devon Bonner, Lorenzo Botto, Brenna Boyd, Lauren C. Briere, Gabrielle Brown, Elizabeth A. Burke, Lindsay C. Burrage, Manish J. Butte, Peter Byers, William E. Byrd, John Carey, Olveen Carrasquillo, Thomas Cassini, Ta Chen Peter Chang, Sirisak Chanprasert, Hsiao-Tuan Chao, Gary D. Clark, Terra R. Coakley, Laurel A. Cobban, Joy D. Cogan, Matthew Coggins, F. Sessions Cole, Heather A. Colley, Cynthia M. Cooper, Heidi Cope, Rosario Corona, William J. Craigen, Andrew B. Crouse, Michael Cunningham, Precilla D’Souza, Hongzheng Dai, Surendra Dasari, Joie Davis, Jyoti G. Dayal, Esteban C. Dell'Angelica, Katrina Dipple, Daniel Doherty, Naghmeh Dorrani, Argenia L. Doss, Emilie D. Douine, Dawn Earl, David J. Eckstein, Lisa T. Emrick, Christine M. Eng, Marni Falk, Elizabeth L. Fieg, Paul G. Fisher, Brent L. Fogel, Irman Forghani, William A. Gahl, Ian Glass, Bernadette Gochuico, Page C. Goddard, Rena A. Godfrey, Katie Golden-Grant, Alana Grajewski, Don Hadley, Sihoun Hahn, Meghan C. Halley, Rizwan Hamid, Kelly Hassey, Nichole Hayes, Frances High, Anne Hing, Fuki M. Hisama, Ingrid A. Holm, Jason Hom, Martha Horike-Pyne, Alden Huang, Sarah Hutchison, Wendy Introne, Rosario Isasi, Kosuke Izumi, Fariha Jamal, Gail P. Jarvik, Jeffrey Jarvik, Suman Jayadev, Orpa Jean-Marie, Vaidehi Jobanputra, Lefkothea Karaviti, Shamika Ketkar, Dana Kiley, Gonench Kilich, Shilpa N. Kobren, Isaac S. Kohane, Jennefer N. Kohler, Susan Korrick, Mary Kozuira, Deborah Krakow, Donna M. Krasnewich, Elijah Kravets, Seema R. Lalani, Byron Lam, Christina Lam, Brendan C. Lanpher, Ian R. Lanza, Kimberly LeBlanc, Brendan H. Lee, Roy Levitt, Richard A. Lewis, Pengfei Liu, Xue, Zhong Liu, Nicola Longo, Sandra K. Loo, Joseph Loscalzo, Richard L. Maas, Ellen F.

Macnamara, Calum A. MacRae, Valerie V. Maduro, AudreyStephannie Maghiro, Rachel Mahoney, May Christine V. Malicdan, Laura A. Mamounas, Teri A. Manolio, Rong Mao, Kenneth Maravilla, Ronit Marom, Gabor Marth, Beth A. Martin, Martin G. Martin, Julian A. Martínez-Agosto, Shruti Marwaha, Jacob McCauley, Allyn McConkie-Rosell, Alexa T. McCray, Elisabeth McGee, Heather Mefford, J. Lawrence Merritt, Matthew Might, Ghayda Mirzaa, Eva Morava, Paolo Moretti, John Mulvihill, Mariko Nakano-Okuno, Stanley F. Nelson, John H. Newman, Sarah K. Nicholas, Deborah Nickerson, Shirley Nieves-Rodriguez, Donna Novacic, Devin Oglesbee, James P. Orengo, Laura Pace, Stephen Pak, J. Carl Pallais, Christina G.S. Palmer, Jeanette C. Papp, Neil H. Parker, John A. Phillips III, Jennifer E. Posey, Lorraine Potocki, Barbara N. Pusey Swerdzewski, Aaron Quinlan, Deepak A. Rao, Anna Raper, Wendy Raskind, Genecee Renteria, Chloe M. Reuter, Lynette Rives, Amy K. Robertson, Lance H. Rodan, Jill A. Rosenfeld, Natalie Rosenwasser, Francis Rossignol, Maura Ruzhnikov, Ralph Sacco, Jacinda B. Sampson, Mario Saporta, Judy Schaechter, Timothy Schedl, Kelly Schoch, Daryl A. Scott, C. Ron Scott, Vandana Shashi, Jimann Shin, Edwin K. Silverman, Janet S. Sinsheimer, Kathy Sisco, Edward C. Smith, Kevin S. Smith, Lilianna Solnica-Krezel, Ben Solomon, Rebecca C. Spillmann, Joan M. Stoler, Kathleen Sullivan, Jennifer A. Sullivan, Angela Sun, Shirley Sutton, David A. Sweetser, Virginia Sybert, Holly K. Tabor, Queenie K.-G. Tan, Amelia L. M. Tan, Mustafa Tekin, Fred Telischi, Willa Thorson, Cynthia J. Tifft, Camilo Toro, Alyssa A. Tran, Rachel A. Ungar, Tiina K. Urv, Adeline Vanderver, Matt Velinder, Dave Viskochil Tiphanie P. Vogel, Colleen E. Wahl, Melissa Walker, Stephanie Wallace, Nicole M. Walley, Jennifer Wambach, Jijun Wan, Lee-kai Wang, Michael F. Wangler, Patricia A. Ward, Daniel Wegner, Monika Weisz Hubshman, Mark Wener, Tara Wenger, Monte Westerfield, Matthew T. Wheeler, Jordan Whitlock, Lynne A. Wolfe, Kim Worley, Changrui Xiao, Shinya Yamamoto, John Yang, Zhe Zhang, Stephan Zuchner

Materials and Methods

Patient Selection

Patients accepted for evaluation in the UDN study typically have previously undergone comprehensive evaluations and testing that have not led to a diagnosis and have overt phenotypic features or family history that suggest a novel disease process. In collaboration with the UDN Clinical Site at Harvard Medical School, we selected a subset of these patients who had presentations that were suspicious for having a component of immune dysregulation (Table S1). Clinical and laboratory characteristics that prompted inclusion into our study included elevated inflammatory markers without alternative explanation, evidence for inflammatory infiltrate in one or more organs, or the presence of autoantibodies with a clinical phenotype that did not match the clinical disease typically associated with that autoantibody. 17 UDN patients were initially accepted for evaluation, though 1 sample could not be analyzed due to insufficient cells available for analysis after mass cytometry data acquisition. SLE patients enrolled in this study met the 1997 American College of Rheumatology (ACR) classification criteria for SLE (1) as well as the 2012 Systemic Lupus International Collaborating Clinics (SLICC) criteria for SLE and the 2019 European Alliance of Associations for Rheumatology (EULAR)/ACR classification criteria for SLE (2, 3). RA patients who were enrolled met the 2010 ACR/EULAR classification criteria for RA (4). For RA patient samples processed for RNA sequencing, PBMCs were collected from 28 patients at two time points.

Study Approval

UDN patients were enrolled with informed consent under NIH IRB Protocol 15HG0130. SLE patients, RA patients, and non-inflammatory controls whose PBMCs were used for mass cytometry were enrolled at Brigham and Women's Hospital with informed consent under IRB protocols 2014P002558 and 2016P001660, which were approved by the Mass General Brigham

IRB. RA patients whose PBMCs were used for scRNA-sequencing were enrolled with informed consent under IRB Protocol 2019P001807.

Mass Cytometry

Blood samples were collected using heparin tubes, and density centrifugation with Ficoll-Hypaque was used to isolate PBMCs. After washing the PBMCs with PBS, samples were cryopreserved in a solution containing 90% fetal bovine serum (FBS) (Life Technologies) and 10% DMSO. We analyzed PBMCs from 16 UDN patients with inflammatory conditions plus 98 non-inflammatory controls, 24 SLE patients, and 20 RA patients as comparators using two panels that were T cell and B cell focused (Table S2). For mass cytometry analysis, cryopreserved PBMCs were thawed and processed in 9 batches, each containing both patients with active disease and controls. In total, there were 5 batches of RA patients and controls processed sequentially, 3 batches of SLE patients and controls processed sequentially (5), and 1 batch of UDN patients and controls. PBMCs were thawed into RPMI Medium 1640 (Life Technologies #11875-085) containing with 5% heat-inactivated FBS (Life Technologies), 1 mM GlutaMAX (Life Technologies), antibiotic-antimycotic (Life Technologies), 2 mM MEM non-essential amino acids (Life Technologies), 10 mM HEPES (Life Technologies), 2.5 x 10-5 M 2-mercaptoethanol (Sigma-Aldrich), 20 units/mL sodium heparin (Sigma-Aldrich), and 25 units/mL Benzonase® Nuclease (Sigma-Aldrich). 500,000 to 1 million cells were taken from each sample. After centrifugation and aspiration of the media, cisplatin viability staining reagent (Fluidigm) at a 5 μM concentration was added for 2 minutes and then the samples were diluted with the culture media. The cells were centrifuged and then blocked for 10 minutes using Human TruStain FcX Fc receptor blocking reagent (BioLegend) diluted 1:100 in media containing bovine serum albumin (Sigma Aldrich) and sodium azide (Sigma Adrich). The cells were then incubated for 30 minutes with conjugated surface antibodies at the indicated dilutions (Table S2). All antibodies were obtained from the Harvard Medical Area CyTOF Antibody

Resource and Core (Boston, MA). Cells were then fixed in 4% paraformaldehyde (PFA) (Fisher Scientific) for 10 minutes, followed by permeabilization using the FoxP3/Transcription Factor Staining Buffer Set (ThermoFisher Scientific). The samples were barcoded by incubation with SCN-EDTA coupled palladium based barcoding reagents for 15 minutes. The samples were all combined into a single sample, and then incubated with conjugated intracellular antibodies for 30 minutes. 1.6% formaldehyde was then used to fix the cells in a 10-minute incubation. Cells were incubated in an iridium intercalator solution (Fluidigm) at 18.75 μM for 20 minutes to label the DNA. Samples were washed and EQ™ Four Element Calibration Beads (Fluidigm) mixed in Milli-Q filtered distilled water was added to the cells for at a dilution of 1 million cells/mL. Samples were analyzed on the Helios CyTOF Mass Cytometer (Fluidigm).

Mass cytometry data processing

Bead-based normalization was performed on the raw mass cytometry files from UDN, SLE, RA and non-inflammatory patients as previously described (6) using the premessa R package (Parker Institute for Cancer Immunotherapy). Files were then analyzed and cleaned to removed debris and doublets through gating in FlowJo™. The files were then processed through the R package CATALYST (7) to perform debarcoding and compensation. Viable PBMCs were manually gated using FlowJo™, and FCS files were created for each sample containing only the PBMCs. Samples containing fewer than 5000 PBMCs were excluded from further analysis. The resultant FCS files along with the metadata were formatted to build a Seurat object (8). We performed principal components analysis, downsampling to 5000 PBMCs per sample, Harmony batch correction (9), clustering (resolution = 0.6), and UMAP visualization using Seurat (8). In the Seurat analysis of samples stained with the T cell marker mass cytometry panel, data on the expression of CRTH2/CX3CR1 and TIGIT were excluded due to inadequate antibody staining. We identified patients who appeared as unique individual outliers by assessing the frequency of the cluster as a proportion of total PBMCs or as a proportion of the immune cell subtype of T

cells, B cells or monocytes and NK cells. We defined an extreme upper outlier as a case in which the single highest frequency of a cluster for a particular patient is greater than 2-fold higher than the next highest cluster frequency with a threshold cluster size for the patient at 250 cells or greater, representing 5% of the patient's total PBMCs.

Single cell RNA sequencing

For CITEseq analysis of UDN #1, $CD4^+$ T cells were selected from cryopreserved PBMCs collected at baseline, after abatacept, and after tofacitinib using the $CD4^+$ T Cell Isolation Kit (Miltenyi Biotec). After incubating the cells with an Fc receptor blocking solution, Human TruStain FcX™ (BioLegend), in Cell Staining Buffer (BioLegend) for 10 minutes at 4ºC, hashing antibody staining was performed for 30 minutes at 4ºC. The cells were washed three times with Cell Staining Buffer and 100,000 cells from each time point sample were mixed. Cells were then stained with TotalSeq™-C Human Universal Cocktail (BioLegend) for 30 minutes at 4ºC, washed 3 times, and resuspended in 0.4% BSA in PBS.

For CITEseq analysis of RA patients, PBMCs were incubated in Human TruStain FcX (BioLegend) for 10 minutes at 4ºC and stained with the LIVE/DEAD™ Fixable Aqua Dead Cell Stain Kit (ThermoFisher Scientific). The BD FACSAria™ Fusion Flow Cytometer was used to sort 100,000 live lymphocytes. Cells were stained with hashing antibodies and the TotalSeq-C Universal Cocktail (BioLegend) as described above for CITEseq of UDN #1.

After counting live cells in 0.4% BSA in PBS using Trypan blue, a maximum of 30,000 cells were loaded onto a Chromium Next GEM Chip G (10x Genomics). cDNA and library generation were performed according to the manufacturer's protocol. mRNA libraries were sequenced using the Novaseq S4 flow cell (Illumina). CITE-seq antibody-derived tag (ADT) libraries were sequenced using the Hi-Seq X Ten System (Illumina). mRNA and ADT unique molecular

identifier (UMI) counts were quantified using Cell Ranger v6.1.1. The FASTQ files were aligned to the GRCh38 human reference genome using Cell Ranger v6.1.1 (10X Genomics). Gene and ADT reads were quantified simultaneously using Cell Ranger count. The files were then processed through the R package Seurat 4.0.6 (8). After normalizing expression of each hashing antibody with the centered-log ratio (CLR), demultiplexing was performed with HTODemux function using positive.quantile = 0.99 (10). Doublets and cells that did not exhibit hashtag staining were excluded.

For the QC filter, we removed cells that expressed fewer than 750 genes, more than 4000 genes, or contained more than 10% of total UMIs associated with mitochondrial genes. After the QC process, we normalized mRNA expression using log-normalize (scale.factor = 10,000) and normalized protein expression with CLR. Then, the top 2,000 most highly variable genes based on a variance stabilizing transformation were selected and scaling was performed. After calculating 20 principal components (PCs) based on the mRNA data, samples were batch corrected with the Harmony R package (9). UMAP visualization and clustering (resolution = 0.5) were performed. We performed pathway enrichment analysis using the ReactomePA package (11). For the top 3 enriched pathways in the Treg cluster, the module scores based on the genes listed in the Reactome pathways (GSEA M983, M705, M965) were calculated with the AddModuleScore function in Seurat. To evaluate T cell activation, we used genes listed in the GSEA Human Gene Set GOBP_T_CELL_ACTIVATION to calculate an activation score using the AddModuleScore function in Seurat. For mapping, we used the mapQuery function in Seurat to map Treg cluster cells of UDN #1 to the reference atlas of heathy donors' peripheral blood data (12).

TCR Repertoire Analysis

TCR Repertoire Analysis was performed with the scRepertoire package (13). After TCR information of each cell barcode was added as metadata with the Seurat object file, cells sharing the same amino acid paired complementarity-determining regions (CDR3) sequences were identified as the same TCR clones. We defined clonality based on number of cells sharing clonotypes: 1 = Single, 2-3 = Small, 4-10 = Medium, 11-30 = Large.

Histology

Formalin-fixed, paraffin-embedded (FFPE) skin biopsies from UDN #1 and a healthy control patient were sectioned at a thickness of 5μm. Sections were separately processed for H&E and immunofluorescence (IF) staining. For sections processed for IF, samples were incubated with the BOND Epitope Retrieval Solution 2 (Leica Biosystems) for 20 minutes at 98ºC. They were then incubated with anti-CD4 antibody diluted 1:400 (DAKO #M7310, Clone 4B12) for 30 minutes at room temperature. Subsequently, the cells were incubated in the Post Primary, Polymer, and Peroxide Block solutions from the BOND Polymer Refine Detection Kit (Leica Biosystems) at room temperature for 10 minutes, 10 minutes, and 5 minutes, respectively. Cells were then incubated with the Alexa Fluor™ 488 Tyramide Reagent (ThermoFisher Scientific) for 5 minutes at room temperature. The sections were then incubated in the BOND Epitope Retrieval Solution 2 (Leica Biosystems) for 10 minutes at 98ºC and anti-FoxP3 (Cell Signaling Technology #98377, Clone D2W8E) at a dilution of 1:50. Samples were incubated with the Polymer and Peroxide Block solutions from the BOND Polymer Refine Detection Kit (Leica Biosystems) at room temperature for 10 minutes and 5 minutes, respectively. They were subsequently incubated in the Alexa Fluor™ 594 Tyramide Reagent (ThermoFisher Scientific) for 10 minutes at room temperature. The sections were incubated again in the BOND Epitope Retrieval Solution 2 (Leica Biosystems) for 10 minutes at 98ºC. Anti-IL2/CD25 antibody (Abcam #ab128955, Clone EPR6452) was applied at 1:250 dilution at room temperature for 30 minutes. Samples were incubated with the Polymer and Peroxide Block solutions from the BOND

Polymer Refine Detection Kit (Leica Biosystems) at room temperature for 10 minutes and 5 minutes, respectively. Samples were incubated in the Alexa Fluor™ 647 Tyramide Reagent (ThermoFisher Scientific) for 10 minutes at room temperature. Samples were then incubated with NucBlue™ Fixed Cell ReadyProbes™ Reagent for DAPI staining (Invitrogen) for 15 minutes at room temperature.

Flow cytometry

Cryopreserved PBMCs were thawed into RPMI supplemented with 10% FBS, washed with PBS, and stained with the LIVE/DEAD™ Fixable Dead Cell Stain Kit (ThermoFisher Scientific). After washing with PBS, the cells were incubated with the following antibodies for 30min at 4ºC: anti-CD3 (BioLegend, Clone UCHT1), anti-CD4 (BioLegend, Clone RPA-T4), anti-CD25 (BioLegend, Clone BC96), anti-CD127 (BioLegend, Clone A019D5). After washing, the cells were resuspended into PBS with 1% BSA. The data were acquired using the BD LSRFortessa™ CellAnalyzer and analyzed by FlowJo™ v10 (Tree Star).

Proliferation suppression assay

$CD4^+$ PI^- $CD25^+$ $CD127^-$ Treg cells were sorted from 3 healthy donors, 3 RA donors, and UDN #1 with the aforementioned flow cytometry antibodies using the FACSAria™ Fusion cell sorter and labeled with CellTrace Violet diluted 1:1000 (Invitrogen) at 37ºC for 15 minutes. $CD4^+$ T cells from a separate healthy donor were enriched using the $CD4^+$ T Cell Isolation Kit (Miltenyi Biotec) and were labeled with CellTrace CFSE diluted 1:1000 (Invitrogen) at 37ºC for 15 minutes. These labeled cells were mixed in a 1:1 ratio (20,000 cells each) in RPMI/10% FBS/L-Glu/0.01 M HEPES with penicillin-streptomycin in 96 U-bottom well plates and stimulated with Dynabeads™ Human T-Activator CD3/CD28 at a ratio of 1:5 bead:cell. After 4 days, the data were acquired using the BD FACSCanto™ II Flow Cytometer. The proliferation of CFSE+

responder T cells was assessed by FlowJo™ v10 (Tree Star). Percent suppression was calculated using standard methods (14).

Western blot

100,000 PBMCs were resuspended with 10 µL of 4X Laemmli sample buffer (Bio-Rad) and mixed with 2uL DTT (1M), 28 µL of dH2O. After incubating lysates for 5 minutes at 95ºC, the samples were loaded onto a 10% Criterion TGX Midi Precast Gel (Bio-Rad) in running buffer (Bio-Rad). After electrophoresis was performed at 120V for 2 hours, the proteins were transferred onto a PVDF membrane at 100V for 1 hour. Then, the membrane was blocked with 5% milk in TBST buffer for 1 hour at room temperature. After washing 3 times with TBST, the membrane was incubated with PSTPIP1 antibody diluted 1:100 (Santa Cruz Biotechnology #sc390727, Clone B-10) and GAPDH antibody diluted 1:5000 (Abcam #ab9485) in TBST at 4ºC overnight. After washing in TBST, the membrane was incubated with HRP-conjugated secondary antibodies each diluted 1:10,000 (Invitrogen #G21234, Invitrogen #31430) in TBST. After washing, the membrane was incubated for 5 min with 10 mL of Clarity ECL Substrate (Bio-Rad) and bands were visualized with the ChemiDoc imaging system (Bio-Rad).

ELISA

Serum samples were stored at -80 ºC until analysis. Serum IL-18 levels were assessed using Quantikine ELISA Human Total IL-18/IL-1F4 Immunoassay kit (R&D Systems). First, 50ul of a standard control and serum diluted with 50uL of RD1N diluent were added into a plate coated with an IL-18 capture antibody and incubated for 2 hours at room temperature. After washing three times with wash buffer, 200uL of IL-18 detection antibody was added into the plate and incubated for 1 hour at room temperature. After washing three times, 200uL of streptavidin-HRP was added into the plate and incubated for 30 min at room temperature. After washing three times, 200uL of substrate solution (mixture of hydrogen peroxide and tetramethylbenzidine) was

added into the plate and incubated for 30 min at room temperature. Then, 50uL of stop solution (2N sulfuric acid) was added into the plate and the optical density was measured using a microplate reader (Bio-Rad) set to 450 nm.

Statistics

Individual patients were identified as outliers if the abundance of any individual cluster was the highest abundance detected in the cohort and if the value was twice as high as the next highest individual. This method of outlier detection was more stringent than using a cutoff of 3 standard deviations from the mean.

Supplemental Figure 1: Multiple patients with outlier clusters identified through mass cytometry.

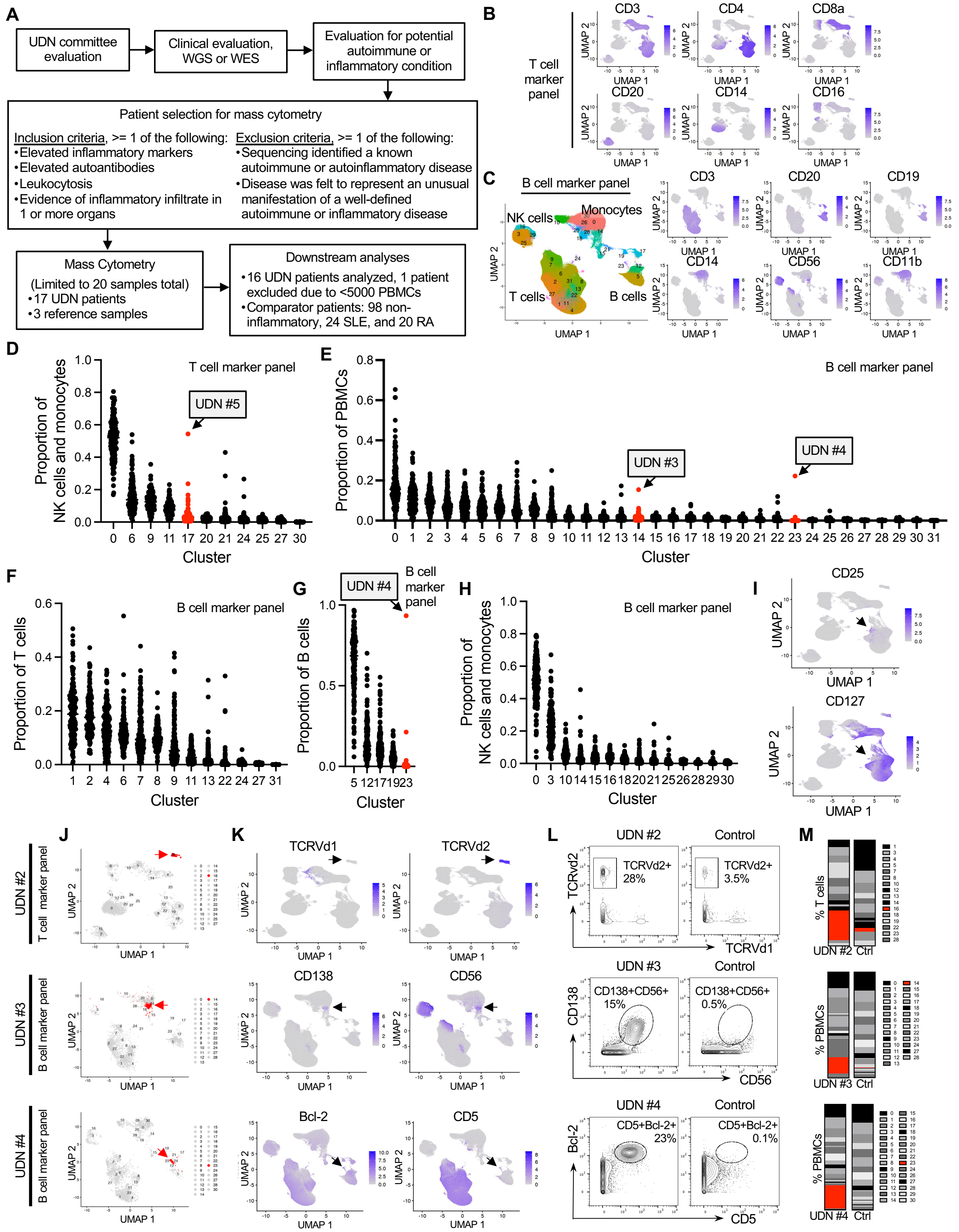

(**A**) Framework and criteria for patient selection. (**B**,**C**) UMAP visualization of major immune cell populations identified in clustering analyses of mass cytometry data from panels consisting of T cell-related markers (**B**) and B cell-related markers (**C**). (**D**-**H**) For all UDN and comparator samples, the frequency of each cluster as a proportion of total PBMCs (**E**) or as a proportion of the indicated cell populations (**D**, **F**-**H**) is depicted as calculated from analysis of the mass cytometry panel consisting of T or B cell-related markers as indicated. In cases where an outlier cluster frequency was detected in a sample, the cluster is depicted in red and the sample name is labeled. (**I**) Feature plots showing expression of CD25 and CD127 in the outlier cluster (arrows) from UDN #1. (**J**) UMAP visualization of outlier cluster (arrows) from individual patients as plotted from T or B cell marker panels as indicated. (**K**) Feature plots depicting major markers that are expressed or absent (arrows) from the outlier cluster in the indicated patient from (**J**). (**L**,**M**) Biaxial gating plots (**L**) and bar charts (**M**) depicted show the frequency of the outlier cluster as a proportion of total T cells for UDN #2 or PBMCs for UDN #3 and UDN #4.

Supplemental Figure 2: UDN #1 exhibits a marked expansion of a regulatory T cell populations and enhanced activation and interferon response signatures.

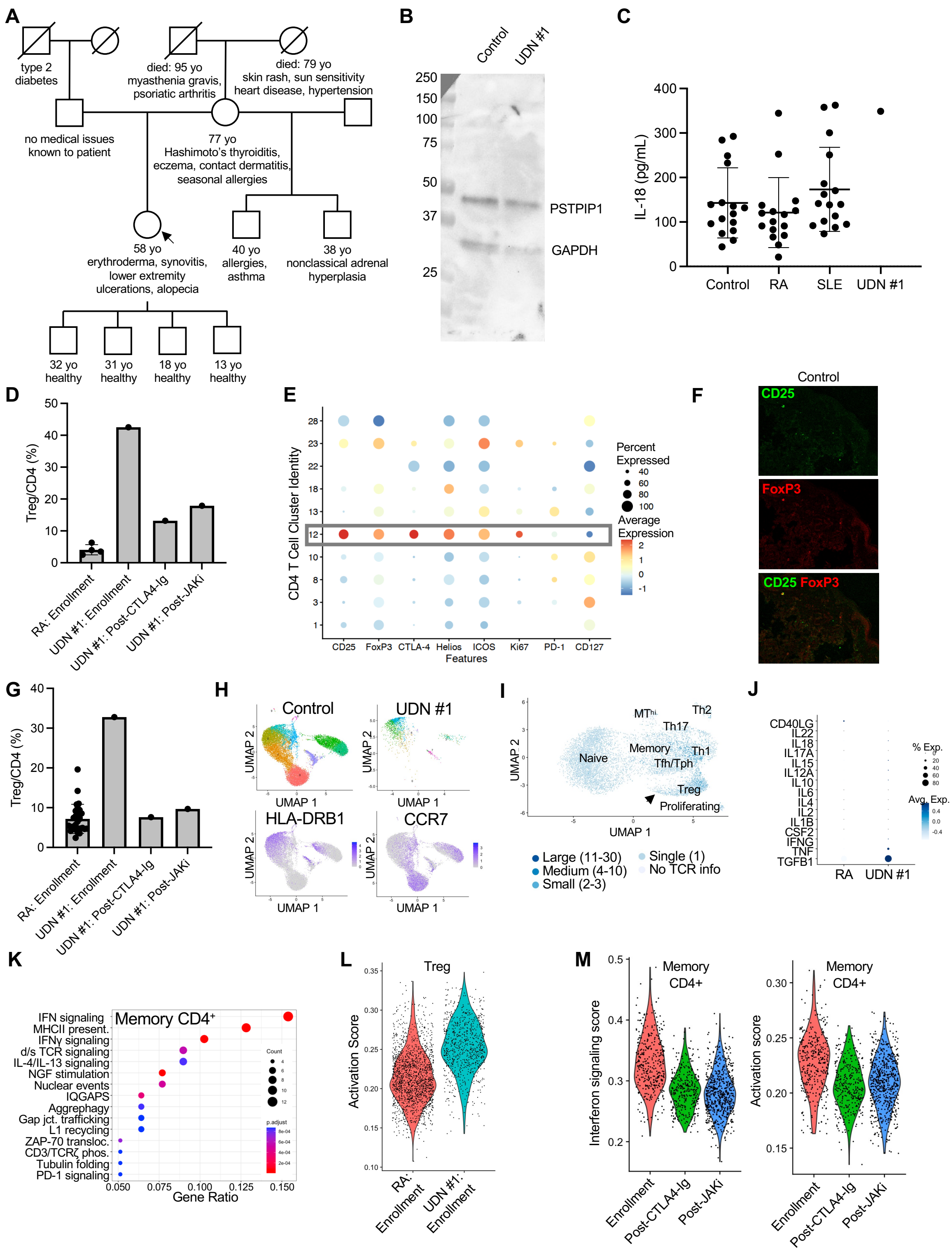

(**A**) Pedigree outlining major medical conditions of UDN #1 (indicated by the arrow) and her family. The patient's father was unavailable for segregation testing but her mother was negative for the variant, meaning the *PSTPIP1* variant is either de novo or paternally inherited. (**B**) Western blot showing the expression of PSTPIP1 and GAPDH in PBMCs from a non-inflammatory control patient and from UDN #1. (**C**) ELISA data showing serum IL-18 levels in UDN #1 and comparator patients including non-inflammatory controls (n=16), SLE patients (n=16), and RA patients (n=16). (**D**) Flow cytometry analysis depicting $CD25^{hi}CD127^{-}$ CD4 T cell numbers as a proportion of total $CD4^{+}$ T cells in RA patients (n=4) and UDN #1 at enrollment, after CTLA4-Ig therapy (abatacept), and after JAK inhibitor therapy (tofacitinib). (**E**) Expression of T cell associated markers in mass cytometry T cell panel among CD4 T cells clusters from UDN #1. The $CD25^{hi}CD127^{-}$ CD4 T cell cluster (Cluster 12) is highlighted in the grey box. (**F**) Immunofluorescence microscopy of skin biopsies from a control patient showing CD25 (green) and FoxP3 (red). (**G**) scRNA-seq data showing regulatory T cell numbers as a proportion of total $CD4^{+}$ T cells in RA patients (n=28) and UDN #1 at enrollment, after CTLA4-Ig therapy (abatacept), and after JAK inhibitor therapy (tofacitinib). (**H**) UMAP of Tregs from UDN patient #1 (top right) mapped to a reference dataset (12) of blood Treg subsets from non-inflammatory control patients (n=8) (top left). Feature plots of HLA-DRB1 and CCR7 expression are shown below. (**I**) UMAP showing frequency of clonotypes in UDN #1 as determined by single cell TCR repertoire sequencing. Treg population highlighted with an arrow. (**J**) Dot plot showing expression of cytokines from scRNA-seq data from RA patients (n=28) and UDN #1. (**K**) Top pathways enriched in memory $CD4^{+}$ T cells from UDN #1 compared to memory $CD4^{+}$ T cells from RA patients (n=28) are listed with gene ratio values depicted from enrichment analysis. (**L**) Violin plots depicting calculated activation scores for Tregs from RA patients (n=28) and UDN #1. (**M**) Violin plots depicting interferon signaling and activation scores in memory $CD4^{+}$ T cells from UDN #1 at enrollment, after CTLA4-Ig therapy, and after JAK inhibitor therapy.

Supplemental Table 1: Clinical Characteristics of UDN Patient Cohort

Patient	UDN ID	Age (years)	Sex	Duration (years)	Clinical Description	Sequencing	Candidate Variant Identified?	Cytometric Finding
UDN #1	UDN 083072	58	F	9	Global erythroderma, anhidrosis, alopecia, and palmar plantar keratoderma. The patient had an inadequate response to multiple immunosuppressive regimens including methotrexate, TNF inhibitors, IL-17 inhibitors, IL-4 inhibitors, and intravenous immunoglobulin.	Duo WGS	Heterozygous variant in *PSTPIP1** gene	Expanded cluster of CD25hi CD127- CD4 T cells comprising 54% of T cells (average = 4.5±4.5%)
UDN #2	UDN 364812	35	F	3	Suspected immune-mediated cerebellar ataxia and extensive white matter disease affecting the frontal lobe, parietal lobe, occipital lobe, brainstem and cerebellum in the setting of low-level GAD65 antibodies (0.04 nmol/L) and isolated N-type voltage-gated calcium channel antibodies (3.57 nmol/L) of unclear significance.	Proband WGS	No	Expanded cluster of Vδ2 T cells comprising 28% of T cells (average = 2.4±3.3%).
UDN #3	UDN 291978	56	M	12	Progressive, symmetric, length-dependent polyneuropathy and relapsing weakness associated with white matter disease not characteristic of multiple sclerosis or other known autoimmune neurologic disease.	Trio WGS	No	Expansion of $CD138^+$ $CD56^+$ monocytes comprising 15% of PBMCs (average = 1.3±1.6%).
UDN #4	UDN 907695	66	M	6	Cranial nerve palsies resulting in dysarthria, dysphagia, diplopia, ptosis, facial muscle weakness, hearing loss, proximal lower extremity weakness and balance problems. The patient had low-level voltage-gated potassium channel antibodies (0.04 nmol/L). A paraneoplastic syndrome was suspected, as the patient exhibited elevated voltage-gated calcium channel P/Q-type (15.0 nmol/L) and N-type (20.9 nmol/L) antibodies. However, extensive evaluation for a primary malignancy, including imaging through PET-CT and other modalities, flow analyses, and bone marrow aspiration and biopsy were unrevealing. Of note, a specified leukemia/flow panel had not been performed. The patient exhibited an inadequate response to multiple immunosuppressive therapies including steroids, intravenous immunoglobulin, plasmapheresis, and rituximab	Proband WES	No	Expanded cluster of $CD5^+$ Bcl-2^+ B cells comprising 22.2% of PBMCs (average = 0.0016±0.0178%). Strikingly, 85.9% of all cells in this unique cluster originated from UDN #4.

UDN #5	UDN 683706	20	F	1	Fevers, myalgias, erythema nodosum and persistent cervical necrotizing lymphadenitis. The patient was refractory to steroid treatment though responded well to adalimumab.	Quad WGS, trio RNA seq	No	Expanded monocyte subset (cluster 16) that could not be readily defined by individual markers.
	UDN 125449	44	F	9	Eosinophilia, chronic inflammatory demyelinating polyneuropathy, multiple allergies and sensitivities, asthma, postural orthostatic tachycardia syndrome.	Proband WES, trio WGS	No	
	UDN 974141	24	F	5-10	Severe chronic sinusitis and atopic dermatitis, unexplained sinus tachycardia, small fiber neuropathy, arthralgias, and myalgias in the setting of marked IgE elevation. Frequent steroid use with partial response of atopic dermatitis to cyclosporine.	Duo WGS, proband RNA seq	Heterozygous variants in *PLA2G7* gene	
	UDN 052220	34	F	8	Persistent enhancing multifocal brain lesions resulting in relapsing sensory and motor deficits. Initially diagnosed with multiple sclerosis but brain pathology not consistent and patient's symptoms and brain lesions progressed despite therapy with steroids, natalizumab, interferon beta-1a, fingolimod, rituximab, cyclophosphamide, and plasma exchange.	Trio WGS	Compound heterozygous variants in *ABCA2* gene	
	UDN 500889	33	M	6	Arthralgias, inflammatory ocular disease, aphthous ulcers, recurrent fevers, splenic necrotizing granulomas, chronic non-specific hepatitis, and gastrointestinal bleeding thought to be secondary to inflammatory lesions. It was felt that the patient's symptoms had features of Behcet's disease and Crohn's disease but were not classic. Though his disease was steroid responsive, he had either no or insufficient response to colchicine, methotrexate, azathioprine, etanercept, adalimumab, anakinra, tocilizumab, and vedolizumab.	Trio WGS, trio RNA seq	Heterozygous variant in *CSMD1* gene	
	UDN 916494	53	F	8	Small fiber polyneuropathy, arthralgias, aphthous ulcers, dry eyes, rashes, angioedema, erythromelalgia, and recurrent thrombophlebitis. Testing notable for low C4 and SSA (Ro) antibody positivity. Medications trialed included methotrexate, adalimumab, abatacept, rituximab, and mycophenolate mofetil. Her disease was responsive to steroids and intravenous immunoglobulin.	Proband WES and RNA seq	Heterozygous variants in *TNFRSF13B* gene	

	UDN 583127	66	M	Since childhood	History of recurrent infections in the setting of mild hypogammaglobulinemia, sensory & motor polyneuropathy, and erythrocytosis among other medical issues.	Proband WES and RNA seq	No	
	UDN 384036	30	F	6	Fevers, arthralgias, muscle weakness, small fiber neuropathy, pancreatic exocrine insufficiency, and livedo reticularis.	Quad WGS, trio RNA seq	Heterozygous variants in *NLRP12* and *RAPGEFL1* genes	
	UDN 287030	34	F	Since childhood	Atypical pervasive developmental disorders in the setting of ornithine transcarbamylase and myeloperoxidase deficiencies, gastrointestinal dysmotility, frequent urinary tract infections, seizure disorder, hiatal hernia, autonomic instability, mast cell activation disorder, and peripheral neuropathy.	Quad WGS, proband RNA seq	Heterozygous variants in *MPO* and *MCM6* genes	
	UDN 753472	32	F	5-10	Eosinophilia, nausea, vomiting, atypical psoriasis, and joint swelling.	Trio WGS, proband RNA seq	Heterozygous variant in *IQGAP1* gene	
	UDN 046412	58	F	12	Moyamoya disease, livedo reticularis, acrocyanosis, and multiple painful lipomas.	Duo WGS	No	
	UDN 462335	47	F	2	Recurrent face, chest, upper back and arm dermatitis.	Proband WGS	Heterozygous variant in *FLG* gene	

WGS, whole genome sequencing; WES, whole exome sequencing; RNA seq, RNA sequencing.

* While the patient did not have a family history of inflammatory disease manifestations (Supplemental Figure 2A), clinical genetic testing identified a novel heterozygous variant c.1222dupG (p.Val408Glyfs*56) in the SH3 domain of an autoinflammatory disease-associated gene PSTPIP1. Because the variant has not been reported in the literature in individuals with PSTPIP1-related disease, it was classified by the clinical reporting lab as a variant of uncertain significance (VUS). Mutations in PSTPIP1 are associated with autoimmune disease, including a spectrum of PSTPIP1-associated inflammatory diseases such as PAPA syndrome, which is an autosomal dominant disorder characterized by pyogenic arthritis, pyoderma gangrenosum, and acne. While the patient's late age of onset and specific clinical features are unusual for PAPA syndrome and other related diseases, we considered whether this mutation could be relevant for disease manifestation. Western blot analysis of PBMCs demonstrated that the size and quantity of the PSTPIP1 protein in UDN #1 was similar to the control individual (Supplemental Figure 2B). Moreover, serum IL-18 levels were not substantially elevated in this patient compared to controls (Supplemental Figure 2C), far less than expected in PSTPIP1 mutation-positive patients with PAPA syndrome (15).

Supplemental Table 2: Mass Cytometry Panels

*Mass cytometry panel below used for all patients except for the following changes for analysis of the RA patient cohort: the CRTH2 antibody was replaced with a CX3CR1 antibody (Clone 2A9-1, Metal 145Nd) in the T cell marker panel and the TRAIL antibody was replaced with a CCR2 antibody (Clone K036C2, Metal 163Dy) in the B cell marker panel.

T cell panel			
**Antigen**	**Clone**	**Metal**	**Dilution**
CD20	2H7	113In	1:100
CD3	UCHT1	115In	1:100
CD27	O323	141Pr	1:100
CD45RA	HI100	142Nd	1:100
PD-1	EH12.2H7	143Nd	1:100
CD39	A1	144Nd	1:100
CRTH2*	BM16	145Nd	1:100
CD14	M5E2	146Nd	1:100
CD45RO	UCHL1	147Sm	1:50
CXCR3	G025H7	148Nd	1:100
Granzyme K	GM26E7	149Sm	1:50
CD96	628211	150Nd	1:100
TCRVd1	REA173	151Eu	1:100
CTLA-4	L3D10	152Sm	1:100
CD69	FN50	153Eu	1:100
CXCR5	J252D4	154Sm	1:100
CD4	RPA T4	155Gd	1:100
CD94	DX22	156Gd	1:100
CD16	3G8	157Gd	1:200
CD8a	RPA T8	158Gd	1:100
CD57	HCD57	159Tb	1:200
ICOS	C398.4A	160Gd	1:100
AHR	FF3399	161Dy	1:50
CD56	NCAM16.2	162Dy	1:100
CCR2	K036C2	163Dy	1:100
Ki67	8D5	164Dy	1:100
FoxP3	PCH-101	165Ho	1:200
CD40L	24-31	166Er	1:100
Granzyme B	GB11	167Er	1:400
Helios	22F6	168Er	1:200
PU.1	phpu13	169Tm	1:100
CD127	eBioRDR5	170Er	1:100
TCRVd2	123R3	171Yb	1:100
NKG2D	149810	172Yb	1:100
CD25	M-A251	173Yb	1:100
TIGIT	MBSA43	174Yb	1:100
T-bet	4B10	175Lu	1:100
CCR7	G043H7	176Yb	1:100
HLA-DR	L243	209Bi	1:200

B cell panel			
**Antigen**	**Clone**	**Metal**	**Dilution**
CD20	2H7	113In	1:100
CD3	UCHT1	115In	1:100
CD27	O323	141Pr	1:100
CD11b	M1/70	142Nd	1:100
CD1c	L161	143Nd	1:100
CD24	ML5	144Nd	1:25
CD19	HIB19	145Nd	1:100
CD14	M5E2	146Nd	1:100
CD86	IT2.2	147Sm	1:100
TLR4	HTA125	148Nd	1:100
CD22	HIB22	149Sm	1:100
CD38	HIT2	150Nd	1:200
CD5	UCHT2	151Eu	1:100
Bcl-2	100	152Sm	1:100
TACI	MAB1741	153Eu	1:100
CXCR5	J252D4	154Sm	1:100
CD23	EBVCS-5	155Gd	1:100
CD95	DX2	156Gd	1:100
T-bet	4B10	158Gd	1:100
CD11c	Bu15	159Tb	1:100
CD307d	413D12	160Gd	1:100
CD138	B-A38	161Dy	1:100
CD56	NCAM16.2	162Dy	1:100
TRAIL*	RIK-2	163Dy	1:100
cPARP	D64E10	164Dy	1:100
IgE	MHE-18	165Ho	1:100
CD40	5C3	166Er	1:100
CD10	HI10a	167Er	1:100
IgA	IS11-8E10	168Er	1:100
BAFFR	11C1	169Tm	1:100
PD-L1	29E.2A3	170Er	1:100
IgG	G18-145	171Yb	1:100
IgM	MHM-88	172Yb	1:100
CD21	Bu32	173Yb	1:100
Ki67	8D5	174Yb	1:100
IgD	IA6-2	175Lu	1:100
Perforin	Dg9	176Yb	1:100
HLA-DR	L243	209Bi	1:200

Supplemental References

1. Hochberg MC. Updating the American College of Rheumatology revised criteria for the classification of systemic lupus erythematosus.. *Arthritis Rheum.* 1997;40(9):1725.

2. Petri M et al. Derivation and validation of the Systemic Lupus International Collaborating Clinics classification criteria for systemic lupus erythematosus. *Arthritis Rheum.* 2012;64(8):2677–2686.

3. Aringer M et al. 2019 European League Against Rheumatism/American College of Rheumatology classification criteria for systemic lupus erythematosus. *Ann. Rheum. Dis.* 2019;78(9):1151–1159.

4. Aletaha D et al. 2010 Rheumatoid arthritis classification criteria: An American College of Rheumatology/European League Against Rheumatism collaborative initiative. *Arthritis Rheum.* 2010;62(9):2569–2581.

5. Sasaki T et al. Longitudinal Immune Cell Profiling in Patients With Early Systemic Lupus Erythematosus. *Arthritis Rheumatol.* 2022;74(11):1808–1821.

6. Finck R et al. Normalization of mass cytometry data with bead standards. *Cytom. Part A* 2013;83 A(5):483–494.

7. Chevrier S et al. Compensation of Signal Spillover in Suspension and Imaging Mass Cytometry. *Cell Syst.* 2018;6(5):612-620.e5.

8. Hao Y et al. Integrated analysis of multimodal single-cell data. *Cell* 2021;184(13):3573-3587.e29.

9. Korsunsky I et al. Fast, sensitive and accurate integration of single-cell data with Harmony. *Nat. Methods* 2019;16(12):1289–1296.

10. Stoeckius M et al. Cell Hashing with barcoded antibodies enables multiplexing and doublet detection for single cell genomics. *Genome Biol.* 2018;19(1):1–12.

11. Yu G, He QY. ReactomePA: An R/Bioconductor package for reactome pathway analysis and visualization. *Mol. Biosyst.* 2016;12(2):477–479.

12. Luo Y et al. Single-cell transcriptomic analysis reveals disparate effector differentiation pathways in human Treg compartment. *Nat. Commun.* 2021;12(1). doi:10.1038/s41467-021-24213-6
13. Borcherding N, Bormann NL, Kraus G. scRepertoire: An R-based toolkit for single-cell immune receptor analysis. *F1000Research* 2020;9. doi:10.12688/f1000research.22139.2
14. Mcmurchy AN, Levings MK. Suppression assays with human T regulatory cells: A technical guide. *Eur. J. Immunol.* 2012;42(1):27–34.
15. Stone DL et al. Excess Serum Interleukin-18 Distinguishes Patients With Pathogenic Mutations in PSTPIP1. *Arthritis Rheumatol.* 2022;74(2):353–357.
