# Supplementary figures and images for: High-dimensional immunophenotyping reveals immune cell aberrations in patients with undiagnosed inflammatory and autoimmune diseases
Source: J Clin Invest. 2023 Dec 15;133(24):e169619. doi: 10.1172/JCI169619 (PMC10721141; doi:10.1172/JCI169619)

Control UDN #1

250

150

100

75

50

37

25

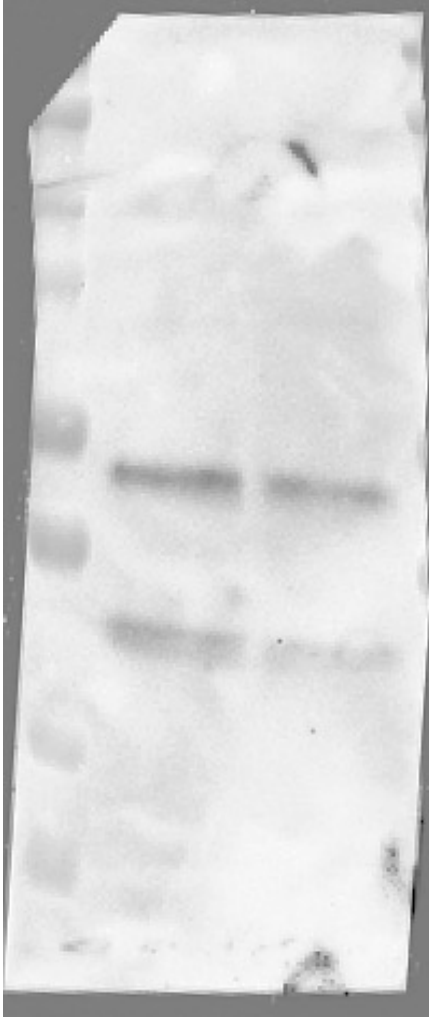

PSTPIP1 (48 kDa)

GAPDH (35.8 kDa)

Supplement: Unedited blot and gel images [file jci-133-169619-s201.pdf]
